# Long-term nutritional trends in the Finnish population estimated from a large laboratory database from 1987 to 2020

**DOI:** 10.1038/s41598-022-09131-x

**Published:** 2022-03-23

**Authors:** Tamara Tuuminen, Mikko Sorsa, Martin Tornudd, Pertti Lauri Lähteenmäki, Tuija Poussa, Pyry Suonsivu, Eeva Marja Pitkänen, Erkki Antila, Kaarlo Jaakkola

**Affiliations:** 1 Medical Center Kruunuhaka Oy, Kaisaniemenkatu 1Ba, Helsinki, Finland; 2 Mineraalilaboratorio Mila Oy, Helsinki, Finland; 3 Solu Digital Oy, Helsinki, Finland; 4 STAT-Consulting, Nokia, Finland

**Keywords:** Health policy, Nutrition, Biochemistry, Biological techniques, Diseases, Medical research

## Abstract

The assessments of malnutrition in adults with MUST or NRS-2002 criteria do not give a detailed insight into the sufficiency of micronutrients. Sufficiency assessment of essential micronutrients on the individual level can be achieved only with laboratory measurements. The aim of this study was to estimate long-term trends in micronutrient sufficiency in the Finnish population with regards to gender and sex covariates. We retrieved from the clinical laboratory database (n = 67,236) all results on whole blood Magnesium, (B-Mg), Manganese (B-Mn), Zinc (B-Zn), Selenium (B-Se) and Copper from erythrocytes (E-Cu) and fasting serum β-carotenes (fS-BKarot), vitamin A (fS-A-vit), coenzyme Q10 (Ubiquinone, fS-Q10) and serum vitamin D (S-D-25) from the database of clinical laboratory Mineraalilaboratorio Mila Oy from the years 1987–2020. A weak positive linear trend is seen for B-Mg, B-Zn and ln(fS-Q10) both for children and adults, but a moderate linear positive trend was observed for ln(S-D-25) based on correlation between calendar year and ln(S-D-25), R = 0.44 and 0.41, p < 0.001 for adults and children, respectively. Laboratory database is helpful to monitor the nutritional public policy to prevent hidden malnutrition in the society.

## Introduction

Malnutrition is a deficiency of not only macronutrients, such as proteins, fat, and carbohydrates but also insufficiency of micronutrients such as vitamins, microelements, co-factor molecules, amino acids, and balanced fatty acids. Deficiency of micronutrients may impair *inter alia* immune and endocrine systems because many micronutrients have bioactive and immunomodulating properties. Macronutrients are the natural carriers of micronutrients. Deficiency of macronutrients may lead to low body mass, loss of muscle mass and disturbed energy supply^1–3^. Persons with obesity are also categorised as malnourished.

There are several ways to study the nutritional status of a person and assess overt or hidden malnutrition. Detection of malnutrition is very important in highly vulnerable patient groups, patients with polymorbidities and chronic diseases such as e.g., cancers^1,3^. Nutritional assessment has become increasingly important in times of CoVID-19 pandemic to predict and prevent poor outcomes and reduce mortality in vulnerable populations^2^. As has been recently outlined, the screening and the assessment of malnutrition in out-patient adults should be initially assessed with the MUST criteria (MUST criteria: see https://www.bapen.org.uk/screening-and-must/must-calculator^2^ or in hospitalized and geriatric patients and especially in polymorbid patients with the NRS-2002 criteria (NRS-2002 criteria: https://www.mdcalc.com/nutrition-risk-screening-2002-nrs-2002^2^. However, these methods are only descriptive screening methods and do not provide a detailed insight into the sufficiency of micronutrients. The only way to estimate the sufficiency of all essential micronutrients is to measure them in the laboratory from patients’ samples.The measurement of micronutrients on the individual level allows performing personalized medicine, to predict and prevent deficiencies and insufficiencies and thus improve the health of the population.High variability of the measurement techniques, reference values, or inconsistency of selection of biological sample materials such as plasma(sera) or whole blood hampers however between-laboratory comparison.

The current literature reviews micronutrient monitoring in several vulnerable population groups, such as e.g. pregnant women^4^ and frailty^5^; in patients with chronic conditions, such as chronic liver diseases^6^, in metabolic syndrome including central obesity, insulin resistance, hypertension, glucose intolerance, and dyslipidemia^7^, the status after bypass bariatric surgery^8^, in conditions related to the bone metabolism^9^, etc. Excessive literature exists to confirm a positive correlation between the micronutrient sufficiency and the resilience to infections^10,11^, the somewhat neglected postulate that needs to be emphasized during the current CoVID-19 pandemics. However, to the best of our knowledge, a retrospective review of the laboratory database to assess nutritional trends in the population from the public health perspective has not yet been published, at least the data covering three decades.

We postulated that laboratory data accumulated during 1987–2020 from our out-patients may reflect the trends in the general Finnish population. The primary aim of this study was to estimate long-term trends in of the levels of micronutrients through the laboratory data acquisition. The secondary aim was to estimate whether age or gender is associated with the level of the micronutrient variables.

## Results

This database includes a total of 67,236 samples from 1987 to 2020. Most samples were from females 45,696 (68%) and 21,540 (32%) samples from males. Median age of male and female subjects was 51 years (range 1–96 years). From adults (age ≥ 18 years) there were 63,113 (94%) samples and from children (age < 18 years) there were 4123 (6%) samples. The majority (59%) of samples was from the age-group 40–69 years.

### Linear trends in micronutrients

**Figure 1:**
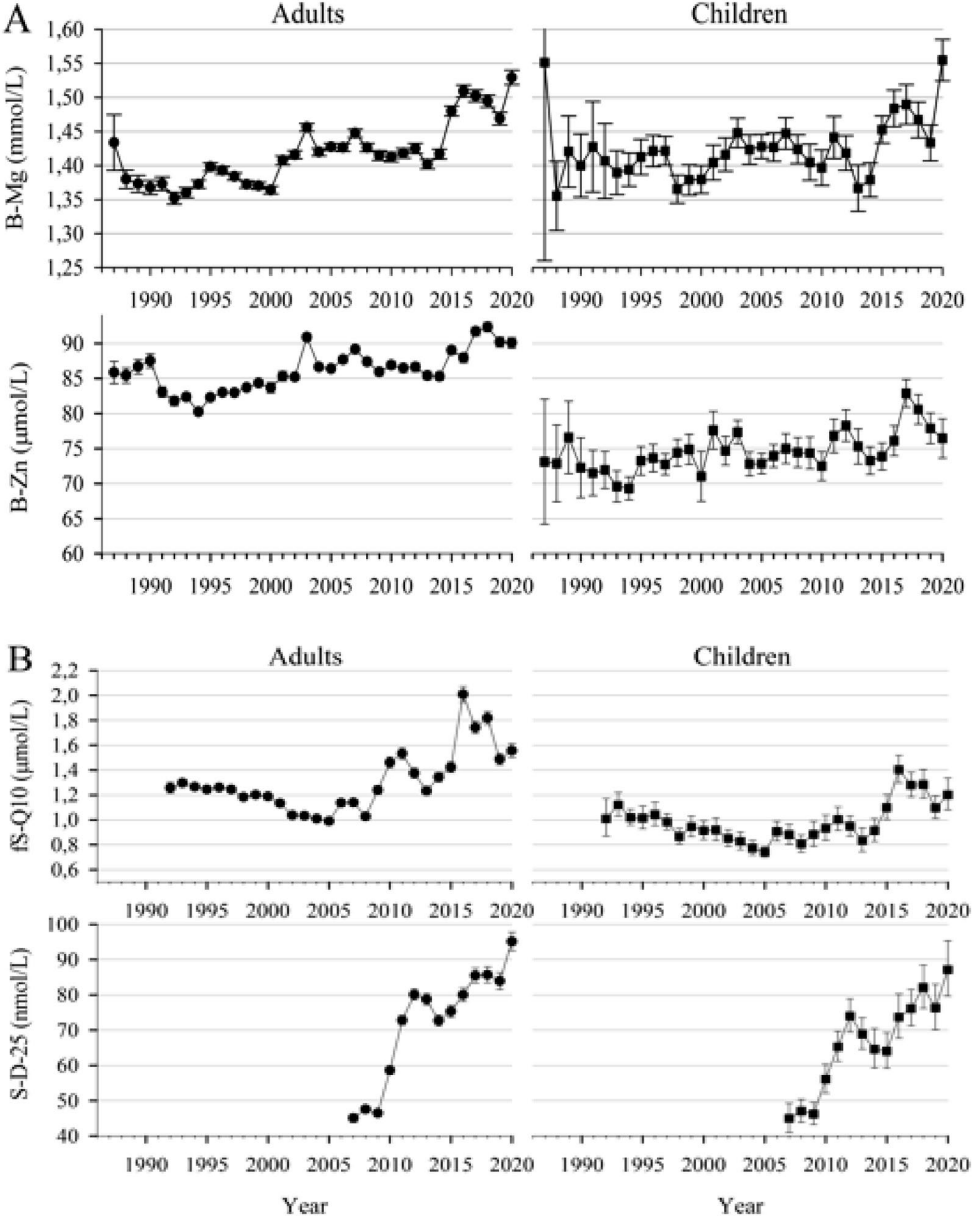
Micronutrient levels in adults (● age ≥ 18) and in children (■ age < 18) measured during 1987–2020. (**A**) Means (95% CI) of B-Mg and B-Zn. (**B**) Geometric means (95% CI) of fS-Q10 and S-D-25. The numbers of results varied from year to year: B-Mg (n = 130–2347 for adults and n = 8–165 for children); B-Zn (151–2523 and 8–201); fS-Q10 (725–1889 and 25–132); S-D-25 (800–1854 and 76–143).

Table1 presents the results of the Pearson s correlation coefficients (R) that assesses a linear trend in the levels of micronutrient during 1987–2020 in adults and in children. Figure 1 illustrates the yearly levels of selected micronutrients.

**Table 1:** Pearson’s correlation coefficients (R) between the calendar year and micronutrients in adults and children.

Year vs. micronutrient	Adults			Children		
	R	P value	N	R	P value	N
B-Mg	0.24	< 0.001	48,372	0.16	< 0.001	2,731
B-Mn	− 0.03	< 0.001	39,433	− 0.02	0.34	2,145
B-Zn	0.19	< 0.001	51,396	0.17	< 0.001	3,452
E-Cu	0.02	< 0.001	42,741	− 0.03	0.10	2,357
fS-A-Vit	0.06	< 0.001	34,802	0.02	0.20	2,616
ln-(B-Se)	0.11	< 0.001	53,464	0.05	0.01	3,396
ln-(fS-Bkarot)	0.01	0.04	32,950	− 0.06	0.005	2,191
ln (fS-Q10)	0.19	< 0.001	39,693	0.14	< 0.001	2,425
ln (S-D-25)	0.44	< 0.001	19,693	0.41	< 0.001	1,586

As seen from the Table 1, all correlations were significant due to the large sample size. A weak positive linear trend is seen for B-Mg, B-Zn and ln(fS-Q10), but the moderate linear positive trend is seen for ln(S-D-25).

Table 2 and Fig. 1 support this observation. Importantly, similar trends were observed for adults and children.

**Table 2:** The slope and the strength (coefficients of determination R^2^) of linear trend for several micronutrients.

	Adults		Children	
	Regression coefficient B (P value)	R^2^	Regression coefficient B (P value)	R^2^
B-Mg	0.004 (< 0.001)	0.06	0.002 (< 0.001)	0.03
B-Zn	0.267 (< 0.001)	0.04	0.227 (< 0.001)	0.03
ln (fS-Q10)	0.012 (< 0.001)	0.03	0.007 (< 0.001)	0.02
ln (S-D-25)	0.057 (< 0.001)	0.19	0.047 (< 0.001)	0.17

R^2^ = coefficient of determination.

It can be implicated from this analysis that e.g., for B-Mg the regression coefficient B of 0.004 means that each year the average B-Mg increased by 0.004 units. The coefficient of determination R^2^ = 0.06 means that 6% of the variance of B-Mg is explained by the year change. All R^2^ values were below 0.20 indicating that the levels of micronutrients were explained mainly by other factors.

### Influence of gender and age on micronutrients

Next, we wanted to test whether the gender or age have a significant relationship with the level of the micronutrient variable. Table 3 illustrates the adjusted means (95% CI) of micronutrients in adult females and males and in age groups ≥ 50 years and 18–49 years, as well as the differences between those groups. Table 4 illustrates the adjusted means (95% CI) of micronutrients in children in age groups 10–17 years and < 10 years, as well as the differences between those groups.

**Table 3:** Adjusted means (95% CI) for micronutrients in adults (age ≥ 18 years) measured between 1987 and 2020 (A: Females vs. males B: Age ≥ 50 years vs. 18–49 years).

A	Females			Males			Females vs. males		
	N	Adj. Mean	95% CI	N	Adj. Mean	95% CI	Adj. Diff	95% CI	P
B-Mg (mmol/L)	32,821	1.396	1.394–1.397	15,551	1.454	1.452–1.457	− 0.059	− 0.062 to − 0.055	< 0.001
B-Mn (μmol/L)	26,212	0.184	0.183–0.185	13,221	0.170	0.168–0.171	0.015	0.013 to 0.016	< 0.001
B-Zn (μmol/L)	35,472	83.9	83.8–84.0	15,924	91.0	90.8–91.2	− 7.1	− 7.3 to − 6.9	< 0.001
E-Cu (mol/L)	28,932	10.12	10.11–10.14	13,809	10.29	10.27–10.31	− 0.17	− 0.19 to − 0.14	< 0.001
fS-A-vit (μmol/L)	23,960	2.04	2.03–2.05	10,842	2.29	2.27–2.30	− 0.25	− 0.27 to − 0.24	< 0.001

	N	GM	95% CI	N	Adj. GM	95% CI	Adj. RGM	95% CI	P
B-Se (μmol/L)	36,815	2.08	2.07–2.09	16,648	2.08	2.07–2.09	1.00	0.99 to 1.01	0.87
fS-BKarot (μmol/L)	21,935	0.85	0.84–0.86	11,008	0.63	0.62–0.64	1.35	1.33 to 1.38	< 0.001
fS-Q10 (μmol/L)	26,633	1.23	1.22–1.24	13,060	1.34	1.33–1.35	0.92	0.91 to 0.93	< 0.001
S-D3-25 (nmol/L)	13,639	68.7	68.2–69.2	6054	66.6	65.9–67.3	1.03	1.02 to 1.04	< 0.001

B	Age 18–49 years			Age ≥ 50 years			Age ≥ 50 years vs. 18–49 years		
	N	Adj. Mean	95% CI	N	Adj. Mean	95% CI	Adj. Diff	95% CI	P
B-Mg (mmol/L)	19,898	1.413	1.411–1.415	28,474	1.437	1.435–1.439	0.024	0.021 to 0.026	< 0.001
B-Mn (μmol/L)	16,059	0.177	0.176–0.178	22,374	0.177	0.176–0.178	− 0.001	− 0.002 to 0.001	0.38
B-Zn (μmol/L)	21,617	86.7	86.5–86.8	29,779	88.3	88.1–88.4	1.6	1.4 to 1.8	< 0.001
E-Cu (mol/L)	17,558	10.29	10.27–10.31	25,183	10.12	10.11–10.14	− 0.16	− 0.19 to − 0.143	< 0.001
fS-A-vit (μmol/L)	15,437	2.11	2.09–2.12	19,365	2.22	2.21–2.23	0.12	0.10 to 0.13	< 0.001

	N	Adj. GM	95% CI	N	Adj. GM	95% CI	Adj. RGM	95% CI	P
B-Se (μmol/L)	22,322	2.02	2.01–2.03	31,142	2.14	2.13–2.15	1.06	1.05 to 1.07	< 0.001
fS-BKarot (μmol/L)	14,274	0.70	0.69–0.72	18,669	0.76	0.75–0.77	1.08	1.06 to 1.10	< 0.001
fS-Q10 (μmol/L)	16,795	1.16	1.15–1.17	22,898	1.42	1.41–1.43	1.22	1.21 to 1.23	< 0.001
S-D3-25 (nmol/L)	8503	64.3	63.7–64.9	11,190	71.1	70.5–71.7	1.11	1.09 to 1.12	< 0.001

Adjustment was based on the general linear model, where age group (≥ 50 years vs. 18–49 years), gender, and 5-year time periods were included as categorical covariates. B-Se, fS-BKarot, fS-Q10 and S-D3-25 were logarithmically transformed before analysis.

*GM* geometric mean, *RGM* ratio of geometric means.

**Table 4:** Adjusted means (95% CI) for micronutrients in children (age < 10 years vs. age 10–17 years) measured between 1987 and 2020.

	Age < 10 years			Age 10–17 years			Age 10–17 years vs. Age < 10 years		
	N	Adj. Mean	95% CI	N	Adj. Mean	95% CI	Adj. Diff	95% CI	P
B-Mg (mmol/L)	1179	1.43	1.42–1.44	1552	1.42	1.41–1.42	− 0.02	− 0.03 to − 0.01	0.001
B-Mn (μmol/L)	953	0.201	0.196–0.205	1192	0.194	0.190–0.198	− 0.006	− 0.011 to − 0.001	0.016
B-Zn (μmol/L)	1585	70.6	70.0–71.2	1867	78.0	77.4–78.7	7.5	6.6 to 8.4	< 0.001
E-Cu (mol/L)	1013	11.23	11.14–11.32	1344	10.78	10.68–10.88	− 0.45	− 0.58 to − 0.32	< 0.001
fS-A-vit (μmol/L)	1201	1.36	1.33–1.39	1415	1.70	1.66–1.73	0.34	0.30 to 0.37	< 0.001

	N	Adj. GM	95% CI	N	Adj. GM	95% CI	Adj. RGM	95% CI	P
B-Se (μmol/L)	1534	1.74	1.72–1.77	1862	1.82	1.79–1.85	1.05	1.02 to 1.07	< 0.001
fS-BKarot (μmol/L)	916	0.75	0.72–0.79	1274	0.60	0.58–0.63	0.80	0.75 to 0.85	< 0.001
fS-Q10 (μmol/L)	1012	1.03	1.00–1.06	1413	0.94	0.92–0.96	0.91	0.88 to 0.94	< 0.001
S-D3-25 (nmol/L)	684	68.5	66.5–70.6	902	60.2	58.6–61.8	0.88	0.84 to 0.92	< 0.001

Adjustment was based on the general linear model, where age group (10–17 years vs. < 10 years) and 5-year time periods were included as categorical covariates. B-Se, fS-BKarot, fS-Q10 and S-D3-25 were logarithmically transformed before analysis.

*GM* geometric mean, *RGM* ratio of geometric means.

Adjustment was based on the general linear model, where age group (≥ 50 years vs. 18–49 years), gender, and 5-year time periods were included as categorical covariates. B-Se, fS-BKarot, fS-Q10 and S-D3-25 were logarithmically transformed before analysis.

The general linear models showed that: (1) For adults and for children and for all nutrient variables there were significant differences when the adjusted 5-year periods were compared (global P < 0.001), (2) Gender had a significant effect on all nutrient variables except on B-Se (Table 3). (3) Age had a significant effect on nutrient variables except on B-Mn in adults (Tables 3 and 4). Importantly, the effects of gender and age were small and were statistically significant due to the large sample size, as seen from the Tables 3 and 4.

### Correlation between the levels of selected micronutrients

**Figure 2:**
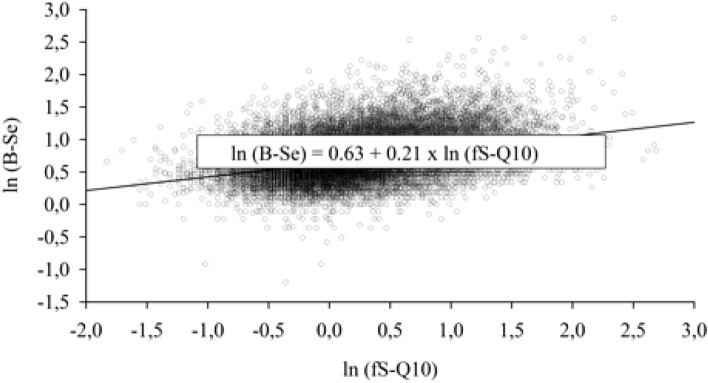
Scatter plot with regression line to illustrate the relationship between fS-Q10 and B-Se (R = 0.36, p < 0.001), both being logarithmically transformed. The results for children and adults are combined (n = 42,118).

Finally, we wanted to test the possible correlation between the levels of selected micronutrients. We selected E-Cu vs. B-Zn; fS-BKarot vs. fS-A-vit and fS-Q10 vs. B-Se because these pairs of micronutrients have either a synergistic effect (fS-BKarot and fS-A-vit, fS-Q10 and B-Se) or are competitors for the absorption in the intestine (E-Cu and B-Zn).The correlations of selected pairs of nutrient variables were R = 0.07 (P < 0.001) for E-Cu vs. B-Zn, R = 0.08 (P < 0.001) for ln(fS-BKarot) vs. fS-A-Vit and R = 0.36 (P < 0.001) for ln(fS-Q10) vs. ln(B-Se). Even though the P values were significant, only the latter correlation indicated a weak linear association. Figure 2 illustrates the correlation between the logarithms of blood selenium levels and serum ubiquinone values.

## Discussion

Here, we present the analysis of the trends in several essential micronutrients measured from our out-patients during the years 1987–2020. We believe that the trends observed in this study can be generalized with some limitations to the Finnish population in general because our patients were not hospitalized but represent mixed population of both genders and all ages. The biggest limitation to this extrapolation is that that individuals with lower income might use less private sector services, therefore their results were underrepresented in this study.

Low grade inflammation is very common and poorly reported in the population in general^12^. In our earlier study^25^ we found that the effect of inflammation on the measurement of β-carotene was dramatic. In situations of severe inflammation, the decline was 90% and in mild inflammation the levels may be halved. Inflammation interacted with the fS-Q10 levels, and the levels of vitamin A. Inflammation was not considered here as a confounding factor because we used a large sample size collected for 34 years. Rather, we were interested in the long-term trends of several micronutrients.

Based on the analysis of this study, we can draw several conclusions: (1) For B-Mg, B-Zn, and ln (fs-Q10) an indication of the positive linear weak trend was detected. (2) A moderate positive trend was observed for ln(S-D-25). (3) This trend was seen for adults and children. (4) There was a significant positive correlation between micronutrients E-Cu vs. B-Zn, between ln(fS-BKarot) vs. fS-A-vit and between ln(fS-Q10) vs. ln(B-Se). However, the large sample size should be taken into consideration and only the last correlation ln(fS-Q10) vs. ln(B-Se) indicated a meaningful positive correlation.

The large sample size used in this study provides an excellent platform to estimate long-term trends in the general population. But there are some pitfalls. Although statistical analysis may point to a significant change these changes are necessary to estimate from the prisms of its clinical significance. The major observation was that over the years the levels of vitamin D has increased in the Finnish population. This is a very welcome trend since the importance of vitamin D sufficiency cannot be overestimated. Unfortunately, today the importance of vitamin D sufficiency has not yet been clearly emphasized by the Finnish public health sector, and it is rarely measured at the routine doctor’s check-up. However, the awareness of the vitamin D immunological and endocrine effects in children and adults^13,14^ has grown among general population due to good access to medical and popular literature and advertisements by the manufacturers. This awareness has grown at the times of the CoVID-19 -pandemic^15^.

Vitamin D is probably the best standardized test across variable techniques. In our laboratory vitamin is measured with HPLC but in other laboratories LC–MS/MS (liquid chromatography-tandem mass spectrometry), chemiluminescence, ECLIA, CLIA or immunoassay are used. According to The External Quality Assessment (EQA) scheme for vitamin D metabolites (DEQAS, https://deqas.org)^16^, some constituents in a sample may have a matrix effect in the ligand binding assays causing inter-sample variability. Matrix does not affect accuracy of the HPLC and LC–MS/MS measurements. It is universally established that the levels < 25, 25–50; 50–75; 75–130 and 375 nmol/L are considered deficient, insufficient, adequate, target values and toxic levels, respectively^13^. Association between low levels of serum vitamin D and increased risk of developing several immune-related diseases, including psoriasis, type 1 diabetes, multiple sclerosis, rheumatoid arthritis, tuberculosis, sepsis, respiratory infection, cancer, and CoVID-19, has been observed^13,17^. However, some individuals might benefit from vitamin D more or less than others as high inter-individual variation in response to vitamin D supplementation has been observed^13^. It should be noted that the levels of vitamin D are affected by seasonal changes being dependent on the solar UV radiation and the exposure to it in latitudes 60–67.5. Therefore, samples collected during the period from September to May reflect primarily nutritional sufficiency than the sun exposure. The results obtained through our study resulting in the average concentration of vitamin D of 68.2–69.2 and 65.9–67.3 nmol/L in females and males, respectively are higher than those reported in the FINRISK Study 1997 Survey performed from February through March 1998 on healthy volunteers. The mean values of vitamin D were 47 ± 34 nmol/L and 45 ± 35 nmol/L (mean ± SD) for females (n = 202) and males (n = 126), respectively^18^. Our results are in accordance with the recently published research data from the Northern Finland Birth Cohort that the level of vitamin D has increased in the Finnish middle-aged population during the period 1997–2013^19^.

Determination of vitamin A, β-carotenes and vitamin E (not studied here) from serum, is usually done by HPLC or UPLC (Scottish STEMDRL https://www.trace-elements.co.uk) or by LC–MS/MS (Mayo Clinic, https://www.mayoclinic.org)). For children, the reference values are usually presented for each age group but for adults the reference values normally are in the range 1.0–3.0 (Scottish STEMDRL) or 1.1–2.7 or 1.1–2.7 (Mayo Clinic). In our laboratory the reference values were calculated as1.4–3.7 and 1.1–3.1 µmol/L for males and females, respectively. Our analysis did not find any trend in the levels of vitamin A, β-carotenes; neither did we found any correlation between the values. This study did not reveal whether there is an insufficiency of these micronutrients in the Finnish population. The importance of vitamin A and β-carotenes is difficult to overestimate. Vitamin A is essential for fetal development and for ocular integrity. The deficiency of vitamin A remains the leading cause of preventable blindness in the world. The deficiency can cause also anemia, and weak resistance to infections. In developed countries, higher consumption of vitamin A, C and E was associated with a significant decreased risk of age-related cataract^20^.

Usually, determination of ubiquinone (Q10) from serum is done by HPLC with electrochemical detection or using LC–MS/MS methodology. Reduced form of ubiquinone is also measured (Mayo Clinic). There are some variations regarding the reference values being0.42–2.50; 0.43–2.55; 0.50–1.77 and 0.48–1.71 µmol/L. There was a slight positive linear trend over the years in the levels of Q10.This trend can be explained by the awareness of the population that the supplementation of Q10 together with selenium may reduce heart complications and improve the overall mental and physical fitness. The four-year prospective placebo-controlled randomized clinical trial found that the overall mortality in elderly has been decreased at the observation time of 12 years^21^.

There are several methods to measure trace elements from biological samples: the AAS (atomic absorption spectroscopy) and the ICP-MS method. The reference values for E-Zn may vary e.g., 67–132 (Labcorp, https://www.labcorp.com) to 153–245 µmol/L (Mayo Clinic). The reference values in our laboratory are 65–107 and 68–115 for females and males, respectively. The reference values for selenium measured from the whole blood is difficult to compare between the laboratories whereas reference levels for B-Mn are similar being approximately in the range 0.06–0.34 µmol/L (for Labcorp, Mayo Clinic, Scottish STEMDRL).

Due to the extremely low Se intake in the 1970s in Finland, an official decision was made in 1984 to supplement multinutrient fertilizers with sodium selenate. Almost all fertilizers used in Finland since 1985 have contained Se^22^. Despite this public health measure, we could not observe any positive linear trend for blood selenium over the years. Instead, only a mild trend was seen for blood magnesium and zinc.

Trace elements and vitamins exert a combined effect on the function of immune system, and their optimamal and balance concentrations against each other is essential to combat virus infections, and current CoVID-19 pandemics in the first place. sufficiency of vitamin A,C, E, and D and zink for the proper function of the immune cells has been recently reviewed^23^.

In conclusion, laboratory measurement of micronutrients is a useful tool to assess not only the nutritional status of individuals but to monitor the nutritional policy for the public health.. Only a weak positive trend from 1987 to 2020 was detected for B-Mg, B-Zn and (ln)fS-Q10, both for children and adults whereas no trend was observed for E-Cu, fS-BKarot, fS-A-vit and B-Se despite its use in fertilizers. To our big satisfaction, a moderate positive trend has been detected for the levels of ln(S-D-25) vitamin in adults and children. These data underline that the usage of laboratory measurement databases is useful for monitoring of nutritional policy among population.

## Materials and methods

### Samples and nutritional variables

For this study we retrieved from the clinical laboratory database all results on whole blood Magnesium, (B-Mg, mmol/L), Manganese(B-Mn, µmol/L), Zinc(B-Zn, µmol/L), Selenium (B-Se, µmol/L) and Copper from erythrocytes (E-Cu, mol/L), the essential microelements, and fasting serum β-carotenes,(fS-BKarot, µmol/L),vitamin A, (fS-A-vit, µmol/L), coenzyme Q10 (Ubiquinone, fS-Q10, µmol/L) and serum vitamin D, (S-D-25, nmol/L). Altogether 67236 results from five out-patient clinics in Finland obtained during 1987–2020 were analysed. We collected data also on gender and age.

The prefaces “fB” refers to a fasting sample, blood; “fS”—the fasting sample, serum; “E” means that the analysis was performed from erythrocytes. The rationale for determination of micronutrients from blood was to accurate estimate the long-term nutrient sufficiency because micronutrients are normally stored in cells, whereas in the serum their concentrations are less stable^4,5^. The concentration of copper is best measured from washed erythrocytes since this minimizes the influence of acute phase reactants^24,25^.

### Analytical methods

E-Cu was analysed with atomic absorption spectroscopy (AAS) from 1987 to 2002, thereafter with inductively coupled plasma atomic emission spectroscopy (ICP-OES) and with ICP-MS (inductively coupled plasma mass spectrometry) from 2014. B-Se and B-Mn were analysed by AAS until 2007 and 2010, respectively. B-Mg and B-Zn were analyzed by ICP-OES until 2014 and then with ICP-MS. B-Mnwere analysed by the in-house acid digestion and the highly sensitive ICP-MS). The ICP-MS is a type of mass spectrometry, where inductively coupled plasma is used to ionize the sample. The sample is atomized, and then atomic and small polyatomic ions are detected (Mineraalilaboratorio Mila Ltd (https://mineraalilaboratoriomila.fi). The values for E-Cu obtained with the new method starting from 2003 were systematically 5% lower than with AAS method, which was acknowledged in the present study. For the other microelements there were no shift in the measured values across the whole observation time.

The fS-BKarot and fS-A-vit are in-house methods (diode array detection (DAD), reverse phase high pressure liquid chromatography (HPLC); fS-Q10 is an in-house method (electrochemical detection, reverse phase HPLC); S-D-25 was detected since 2007 by HPLC.

### Statistical analysis

First, we wanted to answer the primary question whether there is a linear trend towards an increase or a decrease of micronutrient variables during 1987–2010. The data were plotted as a function of time (Fig. 1), the Pearson correlation coefficient (R) was calculated between calendar year and the micronutrient variable, and the regression analysis was performed for adult and children’s samples (Tables 1,2) to illustrate how steeply the increase or decrease happened per one year.

Secondly, the micronutrient variables were analyzed using the general linear model, where age groups (≥ 50 years vs. 18–49 years), gender, and 5-year time periods were included as categorical covariates. We grouped the results into the following 5-year periods: 1987–1990; 1991–1995; 1996–2000; 2001–2005; 2006–2010; 2011–2015 and 2016–2020 because general linear trends were not observed. The age and gender distributions were different in different 5-year periods which might influence the results. The purpose of the linear model and the adjustment was to make the different time points more comparable with each other. Adults and children were analyzed separately. Age group (11–17 years vs. < 10 years) and 5-year time periods were included as categorical covariates in children. In the model’s gender, age group and time were forced into the model as predictors, but the interaction terms were included only if the corresponding p < 0.10. The distributions of the B-Se, fS-BKarot, fS-Q10 and S-D-25 were skewed to the right and were logarithmically (ln) transformed before analyses. The results are given as adjusted means (95% CI) or as adjusted geometric means (95% CI) for both genders, age-categories and for each 5-year period. In addition, the adjusted differences (95% CI) are given for both genders and age groups. For the logarithmically transformed micronutrient variable the ratio of geometric means with 95% confidence interval indicates the relative difference.

Thirdly, the Pearson correlation coefficient (R) was calculated to test the possible correlation between the levels of selected micronutrients: between E-Cu and B-Zn; fS-BKarot and fS-A-vit; fS-Q10 and B-Se. These pairs of micronutrients were chosen because they may have either a synergistic effect or are competitors for the absorption in the intestine.

Due to the very large sample size even very weak correlations and small differences between the groups can become statistically significant. Although a weak correlation may be important in an epidemiological study, here we interpreted the magnitude of a correlation coefficient as the following: 0.00–0.19 a very weak, 0.20–0.39 a weak, 0.40–0.59 a moderate, 0.60–0.79 a strong and 0.80–1.00 a very strong positive correlation.

All statistical tests were two-sided, and p-values < 0.05 were statistically significant. Analyses were performed using IBM SPSS Statistics for Windows (version 27.0, Armonk, NY, USA, IBM Corp.).

### Statements

1. All methods were carried out in accordance with Standard Operational Procedures (SOP) of the laboratory which has got accreditation from the Finnish accreditation service FINAS T 309 (EN ISO/IEC17025). All protocols were approved by the ethical board of Minelaililaboratorio Milia, TK1-28.5.2020
2. Informed consents from all the patients whose blood or sera were analysed during 34 years in Mineraalilaboratorio Mila are irrelevant to this study because: (1) No personal information nor clinical data were used to do this data analysis; (2) The data were analysed blind.

## Abbreviations

AAS: Atomic absorption spectroscopy
A-vit: Vitamin A
B: Blood
BKarot: β-Carotenes
CLIA: Chemiluminescence immunoassay
Cu: Copper
DAD: Diode array detection
D-25: Vitamin D
E: Erythrocytes
ECLIA: Electro-chemiluminescence immunoassay
fS: Fasting serum
HPLC: High pressure liquid chromatography
ICP-OES: Inductively coupled plasma atomic emission spectroscopy
LC–MS/MS: Liquid chromatography-tandem mass spectrometry
Mg: Magnesium
Mn: Manganese
Se: Selenium
Zn: Zinc
Q10: Coenzyme Q10, Ubiquinone
UPLC: Ultra performance liquid chromatography
