# Author Correction: Long-term nutritional trends in the Finnish population estimated from a large laboratory database from 1987 to 2020

**DOI:** 10.1038/s41598-022-15745-y

**Published:** 2022-07-05

**Authors:** Tamara Tuuminen, Mikko Sorsa, Martin Tornudd, Pertti Lauri Lähteenmäki, Tuija Poussa, Pyry Suonsivu, Eeva Marja Pitkänen, Erkki Antila, Kaarlo Jaakkola

**Affiliations:** 1 Medical Center Kruunuhaka Oy, Kaisaniemenkatu 1Ba, Helsinki, Finland; 2 Mineraalilaboratorio Mila Oy, Helsinki, Finland; 3 Solu Digital Oy, Helsinki, Finland; 4 STAT-Consulting, Nokia, Finland

Correction to: *Scientific Reports* 10.1038/s41598-022-09131-x, published online 23 March 2022

Pertti Lauri Lähteenmäki was omitted from the author list in the original version of this Article.

The Author Contributions section now reads:

“T.T. and E.A. designed the study, M.S. retrieved the data, T.P. performed statistical analysis, K.J. was the initiator of the micronutrient measurements in our clinic and together with P.L.L. set the methods in Mineraalilaboratorio Mila Oy. E.M.P. retrieved the data about different techniques and reference methods, M.T. performed the testing and described the techniques used in the laboratory, T.T. wrote the first draft; all the authors contributed equally and approved the final version.”

The original Article has been corrected.

